# Supplementary material for: Differential Expression of BOC, SPOCK2, and GJD3 Is Associated with Brain Metastasis of ER-Negative Breast Cancers
Source: Cancers (Basel). 2021 Jun 15;13(12):2982. doi: 10.3390/cancers13122982 (PMC8232218; doi:10.3390/cancers13122982)
Supplement: Supplementary file 1 [file cancers-13-02982-s001.zip › cancers-1193196-supplementary.pdf]

cancers MDPI

Supplementary materials: Differential Expression of *BOC*, *SPOCK2*, and *GJD3* Is Associated with Brain Metastasis of ER-Negative Breast Cancers

Rute M. S. M. Pedrosa, Leonoor V. Wismans, Renata Sinke, Marcel van der Weiden, Casper H. J. van Eijck, Johan M. Kros and Dana A. M. Mustafa

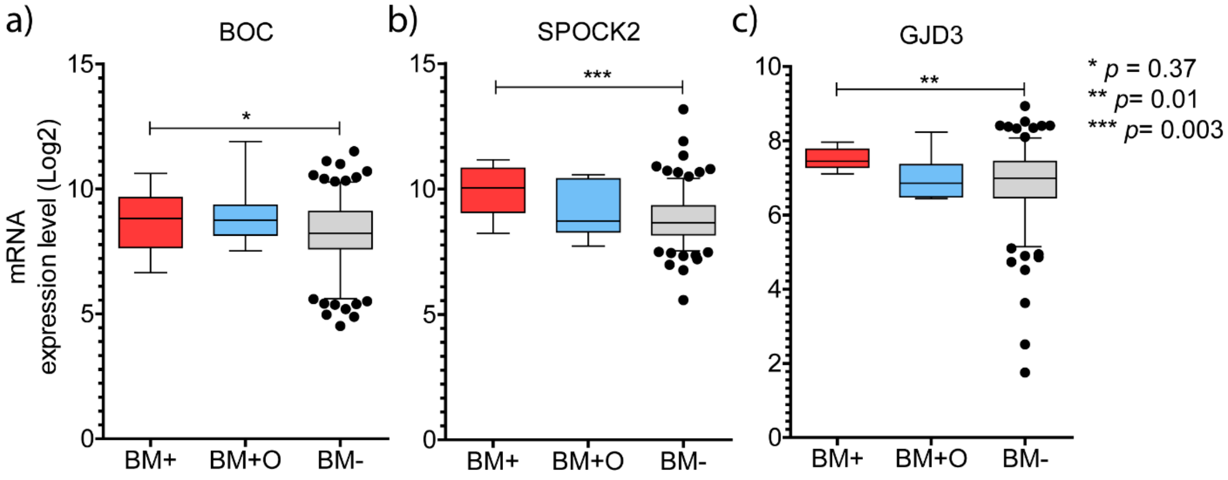

Supplementary Figure S1. (**a**) *BOC*, (**b**) *SPOCK2* and (**c**) *GJD3* mRNA levels in a set of 204 primary breast cancer samples that metastasize to brain (BM+, $n = 8$), to brain and other organs (BM+O, $n = 8$) and primary breast cancer samples that metastasize exclusively to other organs (BM-, $n = 188$). The lowest and highest boundaries of the box represent the 25 and 75 percentiles, respectively. The solid line across the box indicates the median value. Error bars indicate the 5-95 percentile. Dataset information is publicly available under GEO accession number GSE12276, EXP00013 [8].
